# Student perceptions towards online learning in medical education during the COVID-19 pandemic: a mixed-methods study

**DOI:** 10.12688/f1000research.123582.1

**Published:** 2022-08-25

**Authors:** Apurv Barche, Veena Nayak, Arvind Pandey, Ajay Bhandarkar, Shalini G nayak, Kirtana Nayak

**Affiliations:** 1 Medical education, Kasturba Medical College, Manipal Academy of Higher Education, Manipal, Karnataka, 576104, India; 2 Pediatrics department, Kasturba Medical College, Manipal Academy of Higher Education, Manipal, Karnataka, 576104, India; 3 Pharmacology department, Kasturba Medical College, Manipal Academy of Higher Education, Manipal, Karnataka, 576104, India; 4 Anatomy department, Kasturba Medical College, Manipal Academy of Higher Education, Manipal, Karnataka, 576104, India; 5 ENT department, Kasturba Medical College, Manipal Academy of Higher Education, Manipal, Karnataka, 576104, India; 6 Medical and surgical nurisng, Manipal College of Nursing, Manipal, Karnataka, 576104, India; 7 Physiology department, Kasturba Medical College, Manipal Academy of Higher Education, Manipal, Karnataka, 576104, India

**Keywords:** Online teaching, online live lectures, recorded lectures, remote learning, synchronous online learning.

## Abstract

Background: This mixed-methods study was undertaken to ascertain undergraduate medical students’ perceptions of remote learning following the COVID-19 restrictions. 545 students participated in this study. Methods: Data was collected using a validated questionnaire and four focus group discussions. Results: Regarding recorded lectures, the quantitative findings indicated that they were important during online learning and the qualitative findings explained that the recorded lectures enabled individual students to pace and customize their learning. The majority of the students agreed that recorded lectures were relevant to their learning, though they watched less than 50% of recorded lectures. Qualitative findings described procrastination as the rationale for not watching the videos. The online live lectures had a relatively higher percentage of contribution towards learning in comparison with instructor recorded video lectures. Students were more engaged with live lectures, and 63.3% of respondents agreed. Qualitative findings confirmed the opportunities for interacting with peers and better clarification of doubts by teachers during live lectures. Conclusions: Online learning with recorded and live lectures provided continuity in medical education during the COVID-19 pandemic. When compared to recorded video lectures, synchronous live lectures were regarded as superior by students largely due to the opportunity to directly communicate with the instructor and receive quick feedback.

## Introduction

In March 2020, the emergence and outbreak of COVID-19 was declared as a global pandemic by the World Health Organization (WHO) ( Bedford *et al.*, 2020). The Union Ministry of Human resource development and United Grants Commission, Government of India directed nationwide closure of schools and universities and shifted on-campus classes to online modes of teaching ( Times Of India--Online, 2020). The higher educational institutions including medical schools had to explore new tools and technology to reach out to students for uninterrupted curricular delivery to minimize disruption in learning and ensure attainment of required competencies ( Jena, 2020).

Previous studies on online learning formats show that several learning objectives can be achieved virtually with a blend of synchronous and asynchronous methods for remote teaching ( Pei and Wu, 2019). In asynchronous learning formats, resources such as pre-recorded lectures, videos, and interactive learning material with assessments are made available usually through a learning management system (LMS) ( Villatoro *et al.*, 2019). Making portions of curricula asynchronous allows flexibility for individual learners to independently access the content at a time and location of their convenience ( Brady and Pradhan, 2020). Synchronous e-learning requires teachers and students to interact through virtual classrooms at a set time and schedule using technology supporting videoconferencing, live chatting and live streaming ( Redmond, Parkinson, Mullally, 2007). Virtual classrooms with various online platforms can make student-teacher interactions close to the real classroom experience ( Technology, 2014). Online teaching when supplemented with quizzes and self-assessments can make students assess their learning and provide feedback to teachers to make distance education more meaningful ( Ellaway *et al.,* 2008).

Medical education in India before the onset of the pandemic was predominantly classroom-based teaching with face-to-face didactics, small group learning, and hospital-based bedside training for clinical teaching. The use of technology, blended, and hybrid forms of learning remained largely unexplored. Effective online learning requires extensive technological support, and the training of faculty for instructional design and online pedagogy ( Erdem Demiroz, University of Missouri-Kansas City, 2016). Within a short time and as a swift response to the pandemic, health universities shifted to virtual teaching methodologies with minimal faculty training and varying infrastructural capacity (Pattanshetti VM, 2020). Given this context, there is a need for research into the acceptance and impact of online teaching with outcomes from these studies providing insights for blended learning to continue in medical education beyond the pandemic ( National Medical Commission. Module on Online learning and assessment, 2020).

This study is aimed at describing our implementation of online teaching for undergraduate medical students. Various modalities of online teaching-learning methods were made available to the students during the period of COVID-19 restrictions. During the transition phase, pre-recorded lectures were made available to students to access asynchronously and subsequently, online classes were scheduled synchronously in the form of online live lectures. In addition, the assessment of online learning was conducted using quizzes and assignments.

Quantitative and survey-based studies have emerged evaluating the impact of the COVID-19 pandemic on medical students ( Loda *et al.*, 2020; Harries *et al.*, 2021) with the assessment of students attitudes towards remote, distant, and online learning ( Al-Balas *et al.*, 2020; Alsoufi *et al.*, 2020; Shahrvini *et al.*, 2021). But limited literature on qualitative studies ( Khalil *et al.*, 2020) have been published exploring medical students’ perspectives. Hence, this mixed-methods study was undertaken to bring together the quantitative dataset and the complementary strengths from qualitative in-depth data to get a deeper and more nuanced understanding of factors determining student reactions towards remote learning as the only modality available forteaching in an unprecedented situation like COVID-19.

The theoretical framework of Garrison *et al*.’s “model of community of inquiry” (CoI) was used to frame and understand how online learning shapes educational experience among students ( Garrison and Arbaugh, 2007). This model highlights three core elements - cognitive presence, social presence and instructor presence - that guide the design and delivery of online learning experiences. The cognitive presence explains the extent to which the learners would be able to construct and confirm meaning through sustained reflection and critical thinking. Social presence provides the capacity to openly communicate and collaborate to develop a virtual community of learning. The instructor has a key role in the design and delivery of online learning and the teaching presence would integrate cognitive and social processes to support learning. As the primary focus of our study was an educational experience in the context of online learning, the CoI model was adopted to frame the original hypothesis that the factors influencing cognitive, social, and instructor presences and their interaction would drive student perception towards online lectures.

### Program structure

The duration undergraduate medical training program in India is four and half years with a compulsory rotatory internship of one year. This study was conducted at a tertiary care hospital and teaching institute that has an annual enrollment of two hundred and fifty students. The first two years involve coursework in basic sciences with an introduction to community health and forensic medicine. The rotatory clerkships at the hospital with postings to visit rural and community health start from the second year. Year 3, parts 1 and 2 primarily involve learning clinical disciplines with rotatory clerkships. In addition, there is training in attitude, ethics, and communication skills (AETCOM). Until nation-wide COVID-19 restrictions for universities, students were immersed in institutional learning experiences such as lectures, tutorials, sessions in skills lab and rotatory clinical clerkships in the hospital. Field visits were taken up to teach public health and community-based practice. With the imposition of lockdown, students across all professional years moved to their hometown or were confined to the housing facility on the campus.

### COVID-19 academic task force and faculty training

To deliver the curriculum in an online mode, a task force group was set up with Dean as the chairperson and members from the Department of Medical Education for curricular redesign to suit remote delivery. The faculty medical educators underwent the smart teacher training program by the Federation of Indian chambers of commerce and industry for empowering teachers for the digital future and also, online courses for learning to teach online. For training faculty on online teaching, virtual classrooms were created to provide opportunities to explore tools and interactive features to deliver live lectures and also to experience the online class as a student. They were also taught to construct online formative assessments using Microsoft Office Forms and LMS for managing learning resources and to conduct and grade assignments. The students were taught to navigate an online classroom with steps to follow and to practice etiquette when using online classes.

### Online teaching methods

The traditional didactic lectures occurring in classrooms were recorded using an Institutional lecture capturing system (Impartus solutions). During the initial part of the pandemic (4-6 weeks), these recorded lectures from previous batches were made available to students along with PowerPoint slides through institutional LMS. Subsequently for the next 10-12 weeks, after the students and faculty were oriented for teaching and learning deploying virtual classroom software, the lectures were then conducted through an online platform. Impartus virtual classroom for live lectures was utilized first and later Microsoft Teams was used. Quizzes and assignments were held after classes for students to submit using Microsoft Office Forms and Institutional LMS.

## Methods

### Study design

A prospective single-centre cross-sectional study using a mixed-methods research approach was conducted at a university teaching hospital in India. We adopted a convergent design; both quantitative and qualitative data were collected and analyzed during a similar time frame ( Fetters *et al.*, 2013). The quantitative data was collected using a survey in May 2020. The qualitative data was collected by conducting four focused group discussions from May to June 2020. These data were collected in the year 2020 after six months of induction into online teaching.

### Study participants

All students (n=1012, 489 men, 523 women), aged 18-24 years enrolled on the undergraduate medical program who had access to online teaching using live and recorded lectures were invited to participate in the study. The students belonged to all four professional years.

### Data collection

*Quantitative stage*

Quantitative data were collected through an online survey administered through Google Forms. The researchers sent messages about the survey through the student information system and reminders through WhatsApp. Of the 1012 learners, 629 viewed the survey, 627 started the survey and 545 completed it. Thus approximately 87% of students who viewed the survey also completed it and all the submitted responses were complete without any missing items.

*Qualitative stage*

The quantitative data collected were further explored with focus group discussions (FGD1-4). An FGD was held for each professional year and four such FGDs were conducted. Each FGD was comprised of four to five students. The respondents were the student representatives of each professional year with the interviewers being faculty from the Department of medical education. We used nested sampling ( Polit and Beck, 2012) in this study, where the participants in the qualitative strand were a subset of the participants of the quantitative strand.

**Figure 1.**
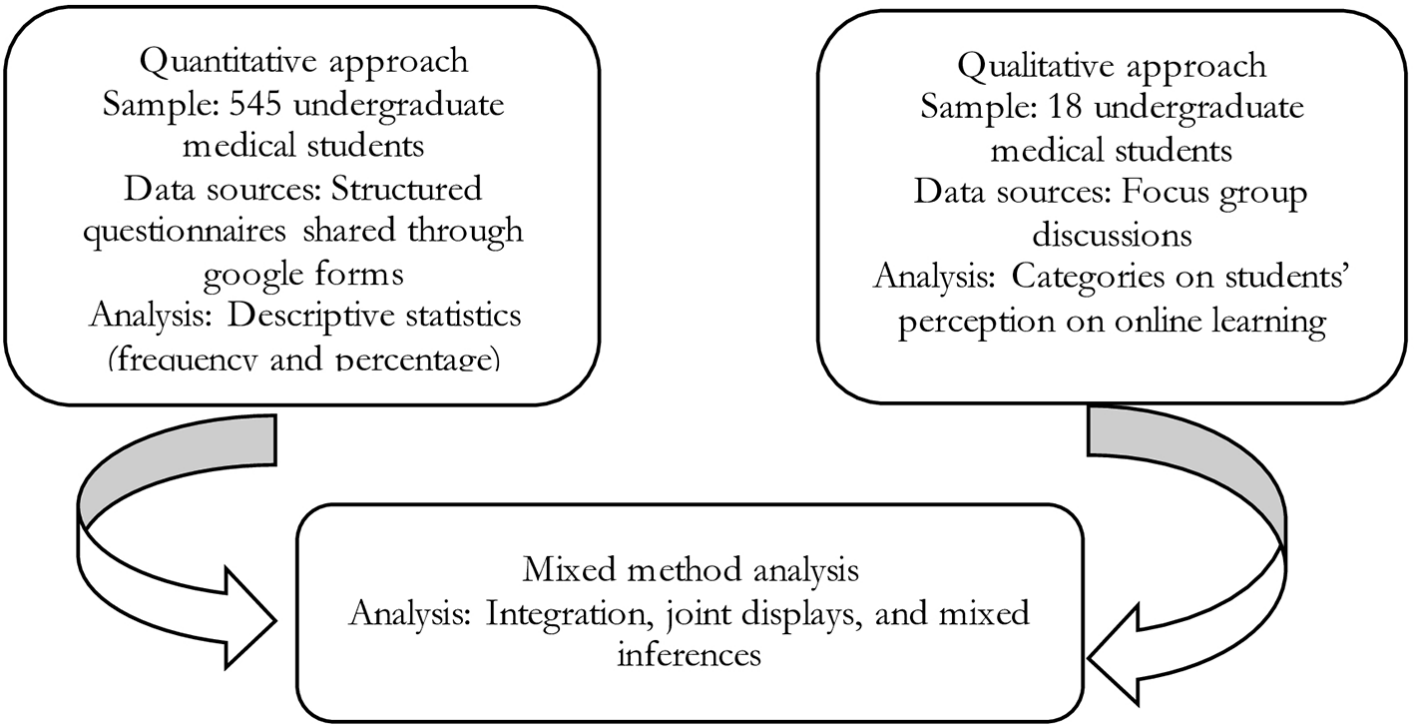
Schematic representation of research design.

We separately analyzed the data from quantitative and qualitative strands. Integration was then done in the mixed-method analysis ( Fetters *et al.*, 2013) and findings were summarized as joint displays ( Guetterman *et al.*, 2015). The research design is depicted in Figure 1.

### Study instruments

*Quantitative stage*

Following an extensive review of literature for online teaching, the questionnaire for the quantitative strand of this study was finalized with the questions presented in two sections. The first section of the questionnaire obtained feedback regarding the usage of online learning and learning resources. The second part included a 13-item validated questionnaire with closed-ended items on a five-point Likert scale ( Dziuban *et al.*, 2015) to measure student satisfaction with online lectures. For the last question, students were asked to use free text to provide any suggestions, ideas, or preferences regarding the use of recorded and live lectures in online classes.

The current version of the 13-item questionnaire is a modified form of the scale for measuring student satisfaction with online learning ( Dziuban *et al.*, 2015). Based on the pedagogical research context, nine items were retained, two item statements (item no. 9 and 13) were modified and two additional items (item no. 10 and 11) were included in the present questionnaire. The scale was validated by seven experts in medical education. Out of the 13 items, 11 items had an item content validity index (I-CVI) of 0.86 while the remaining two items reported an I-CVI of one. All 13 items reported high relevance according to the experts providing a scale content validity index (S-CVI) score of 0.8. The responses of 80 students were analyzed for the internal consistency of the questionnaire. The Cronbach’s alpha for 13 items on students’ perception was found to be 0.89, indicating a good reliability measure for the questionnaire. A copy of the questionnaire can be found under *Extended data* ( Kirtana *et al.*, 2022).

*Qualitative stage*

A semi-structured FGD guide was used to conduct four FGDs. The questions in the guide gathered in-depth data on the perception of students towards online learning with recorded and live lectures. Each FGD lasted for approximately two to two and half hours and the discussions continued to reach the point of data saturation. An online mode was adopted to conduct FGD using Microsoft Teams function.

### Data analysis

*Qualitative data*

The qualitative data collected from FGD and free text responses from the open-ended questions were analyzed by using the framework method. The framework method is a systematic and flexible approach to analyze the qualitative data and an excellent tool for supporting thematic (qualitative content) analysis. This method was most suitable for the analysis of interview data, where it was desirable to generate themes by making a comparison between the cases ( Gale *et al.*, 2013). We have used OpenCode software (OPC) in our analysis as this tool helps for classifying and sorting any kind of qualitative text information. Framework analysis involves seven interconnected stages in multidisciplinary health research: (1) transcription, (2) familiarization with the interview, (3) coding, (4) developing a working analytical framework, (5) applying the analytical framework, (6) charting data into framework matrix and (7) interpreting the data ( Gale *et al.*, 2013). Transcripts were reviewed several times for familiarization and searched for emerging meanings and patterns. Author SGN performed the initial coding and during subsequent coding and analysis, investigator triangulation was used. Analysis was done through the inductive analysis process, without trying to fit the data into preexisting coding frames, themes, or theories. Eight categories related perception of students towards asynchronous and synchronous online learning were identified: Impact of online resources on learning ( *Online teaching supported education continuity during COVID-19)*, importance of recorded videos of lectures ( *Convenient self-paced learning as an advantage for recorded lectures*), viewing of recorded lectures and reasons for not watching recorded lectures ( *Procrastination propensity in recorded lectures*), contribution of live lectures in comparison to recorded lectures for learning ( *Live online lectures are superior*), students’ reaction to live lectures ( *Better clarified concepts in live lecture*), students’ perception towards live lectures ( *Better time management but rigid schedules in live lectures leads to lack of focus*), preference of students towards variety of learning resources and ( *Quizzes and PowerPoints created by teachers supplements learning*), and students’ reaction for online live lecture after lockdown ( *Preferential interest in classroom (face-to-face) learning*). During the analysis, codes and findings were discussed and disagreements were resolved by discussion until consensus was achieved.

*Quantitative data*

Quantitative data were analyzed descriptively with SPSS-16 software. The data were checked for normality. Descriptive statistics were represented as frequency and percentages for the Likert scale responses from the closed-ended questions.

### Mixed-method integration and analysis

Integration is the hallmark in mixed-method analysis and it dramatically enhances the value of research ( Fetters *et al.*, 2013). In integration, the researcher intentionally brings together the quantitative and qualitative data. In our study, the integration occurred at several levels. At the study design level, we used the convergent design to collect quantitative and qualitative data within the same time frame. Initially in analysis, the quantitative and qualitative data were analyzed separately and a more iterative process was followed in the subsequent mixed-method analysis. The findings were also discussed and interpreted in conjunction with mixed-method findings.

We used joint displays to integrate and represent mixed-method analysis as they appear to provide structure to discuss the integrated mixed-method analysis and assist both reader and researcher in understanding how mixed-methods provide new insights ( Guetterman *et al.*, 2015). The joint display contained figures and tables of quantitative variables followed by the corresponding qualitative domain (themes). We opted for *‘fit’ of Data Integration,* which assesses the fit between qualitative and quantitative findings as confirmation, expansion, or discordance. *Confirmation* was achieved if the findings of both data sets confirmed the results of the other, *expansion* if the findings of two sources of data expanded the insights of online mode of learning by explaining its need and strengths, and *discordance* if the findings were contradicting ( Fetters *et al.*, 2013). The qualitative quotes and summaries were arrayed for each domain. We focused on concordance between quantitative and qualitative results.

### Ethical considerations

The study was approved by Kasturba medical college and Kasturba hospital institutional ethics committee (IEC no 33/2020). Informed, written consent was obtained from the participants before completing the survey forms and verbal consent was recorded before the online focus group interviews. The participants were informed that the data would be analysed and shared after complete anonymisation and removal of all identifiable information.

## Results

In the present study, 545 students studying across the professional years of medical school completed the survey. The profile of the students who participated in the study is found in Table 1.

**Table 1.** Students’ profile.

Professional year	Survey response Frequency (%)	Focus group discussion participants Frequency (%)
First-year	137 (25.1)	5 (27.78)
Second-year	114 (20.9)	4(22.22)
Third-year – Part 1	68 (12.5)	4(22.22)
Third-year – Part 2	226 (41.5)	5(27.78)
Total	545 (100)	18 (100)

Results of this study are shown as joint displays. The quantitative findings are shown followed by the qualitative findings and mixed-method inferences.

## Quantitative and qualitative findings on the impact of online lectures on learning during COVID-19 pandemic

### Quantitative findings-1

**Figure 2.**
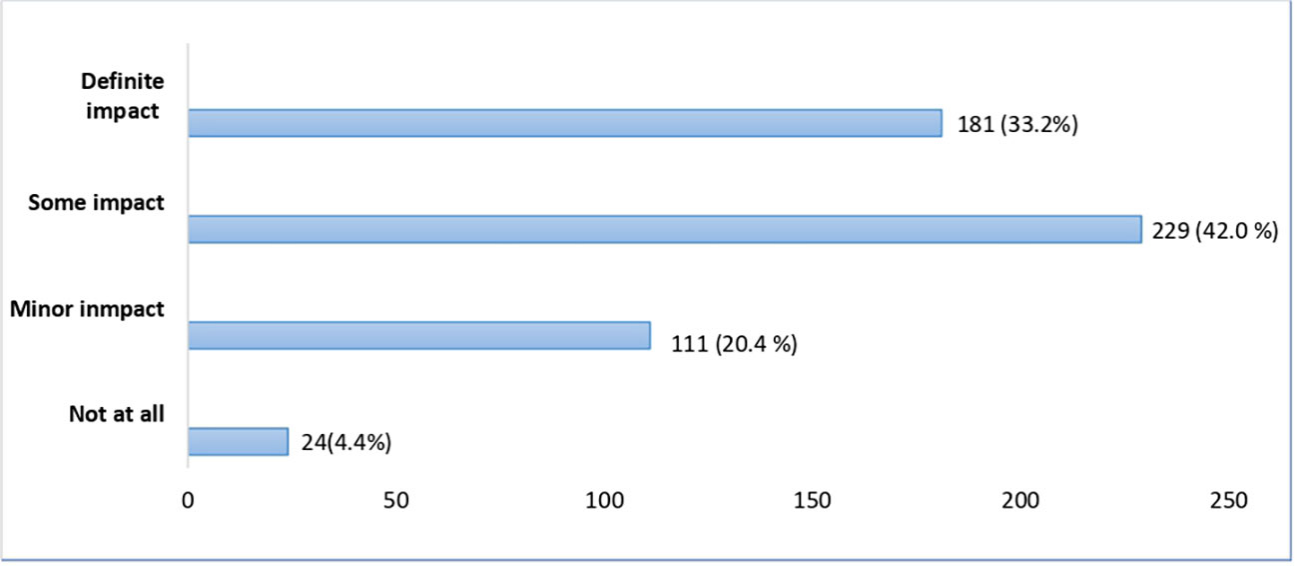
Impact of online resources including live and recorded lectures in learning experience.

In the context of a prolonged closure of the medical school, 75.2% of students responded that online teaching with recorded and live lectures had either definite or some impact on their learning experience. During COVID-19 restrictions, online lectures gave a sense of continuity in teaching and learning as claimed by 353 (64.8%) of students, while 103 (18.9%) disagreed and 89 (16.3%) of them were uncertain about it. The full responses can be found under *Underlying data* ( Kirtana *et al.*, 2022).

### Qualitative findings-1: Online teaching supported education continuity during COVID-19 pandemic

The students contended and were grateful to the teachers and administration for making efforts and finding means for the continuation of education in the context of a prolonged closure of the medical school. Expression of the adequacy of virtual classes was expressed as “ *I am quite happy with what types of classes are happening. We get the live lectures as well as the recorded ones, from a student perspective, what type of classes we are having now, I think it's good. It's good enough for us”* (FGD 1). Similarly, amidst the pandemic during the lockdown, the online classes helped the students maintain the continuity and timely completion of the course, which was expressed, as *“… this is the best thing that can be done during this time for keeping the learning in continuity”* (FGD 2). The full transcripts can be found under *Underlying data* ( Kirtana *et al.*, 2022).

### Mixed-methods inferences 1: Confirmation

Quantitative and qualitative findings on the impact of online classes on continuity of learning complement each other. Quantitative findings indicated that most of the students agreed on the contribution of online classes to the continuity of teaching and learning. Similarly, the qualitative findings also confirmed that online teaching supported the education continuity during COVID-19.

## Quantitative and qualitative findings regarding the importance of recorded lectures

### Quantitative findings-2

**Figure 3.**
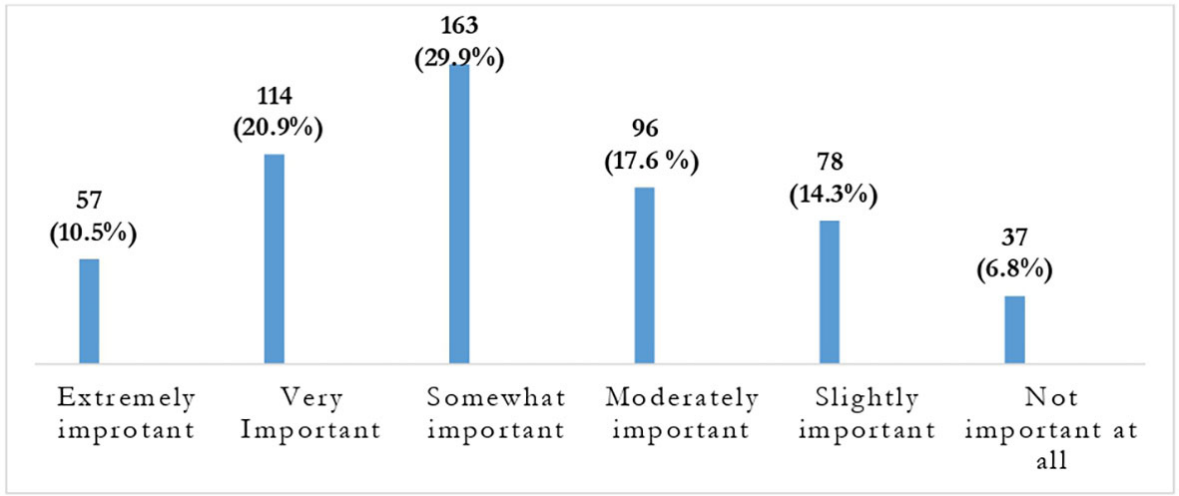
Response of students regarding importance of recorded video lectures.

In the given context, the recorded videos of online lectures in the current course were notably important for 334 (61.3%) of the students. Only 37 (6.8%) of them responded as “not important at all”.

### Qualitative findings-2: Convenient self-paced learning as an advantage for recorded lectures

In the context of lockdown imposed by the COVID-19 pandemic, recorded lectures enabled the individual student to learn without any time pressure which otherwise exists in live online classes. It also enabled the students to learn at their own pace and therefore considered as convenient especially by slow learners. One student each from FGD 1 said, *“Recorded lectures are actually convenient in the way that you could pause them. Check anything if you had a doubt, we watch them again, so it was a really convenient form for me”* (FGD 1). This mode is beneficial for the learners preferring to refer to the learning material several times, enabling note-making and thus enhancing active learning. A student expressed, *“I'm a pretty slow learner. So, for me to understand the concept, I might have to hear it two, three times. So obviously I prefer recorded lectures”; “…it will be easier to make note again, so I clearly prefer recorded”; “…because you can slow it down and you can repeat things once or twice on your convenience”* (FGD 3). The possibility of referring to other resources along with recorded lectures was considered as an advantage. Also, adjusting the speed of recordings was also considered as advantageous. They expressed, *“…we can watch it on 1.5-2x speed, we can pause and rewind which helps me understand concepts then and there, take proper notes”* (Open-ended responses). Distraction related to technical difficulties was perceived as minimal during recorded lectures. One student from FGD2 said, *“I feel better having a little bit more of the benefit because of saving from the technical difficulties and unmuting of other people’s mikes and all that, that acts as an extra distraction in case of the live lecture which is not there in the recorded lectures.”*

### Mixed-methods inferences 2: Expansion

Quantitative and qualitative findings regarding the importance of recorded lectures expanded each other. While quantitative findings indicated that the recorded lectures were important in the current course of learning, the qualitative findings explained how the recorded lectures enabled individual students to pace and customize their learning. The recorded lectures provided considerable flexibility facilitating better comprehension, retention, and focused learning. Thus, convenient self-paced learning in recorded lectures was considered important by the students.

## Quantitative and qualitative findings related to the frequency of viewing of recorded lectures

### Quantitative findings-3

**Figure 4.**
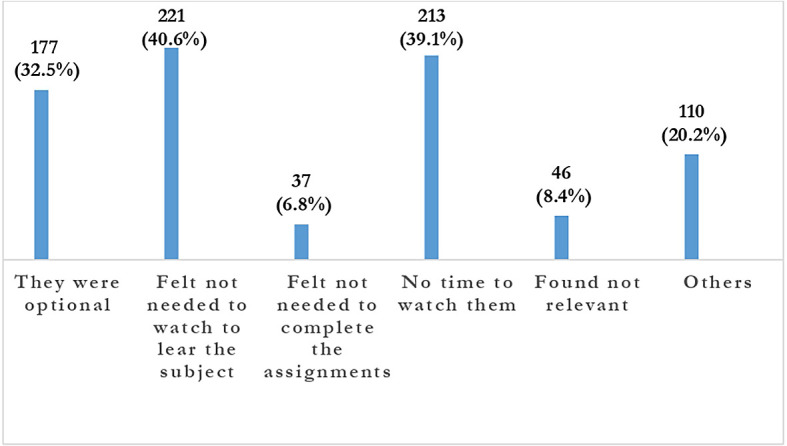
Students response regarding reasons for not viewing recorded video lectures.

The majority of students 648 (91.6%) knew that recorded lectures were relevant for their learning, but most 332 (60.9%) of students watched less than 50% of the recorded lectures. Watching recorded lectures was not made mandatory 177 (32.5%). Recorded lectures were not an absolute requirement to learn the subject 221 (40.6%) and lack of time to watch them 213 (39.1%) were the different reasons cited by students for not watching the recorded lectures.

### Qualitative findings-3: Procrastination propensity in recorded lectures

Students tend to postpone viewing the recorded lectures. The advantage of watching the recorded lectures at one’s own pace may lead to procrastination and may culminate in not viewing the lectures and failure in learning. Students from different FGDs said, *“… there is the tendency to procrastinate if we are given the links because that is what happens honestly…”* (FGD 1); *“… if you have a set of recorded lectures, chances are that you’re probably going to end up like procrastinating and then not do it”* (FGD 2); and *“I feel like since we're at home, a lot of students tend to procrastinate, so we might end up not watching the recorded ones. Even if we had planned to”* (FGD 3).

### Mixed-methods inferences 3: Expansion

The quantitative findings described the frequency and percentage of students watching the recorded lectures and the reasons for not watching. Qualitative findings expanded the reasons for not watching the recorded lectures. Though the majority perceived that the recorded videos were relevant for their learning and even though students desire to watch them, they perceive that the procrastination propensity may culminate in not viewing the lectures. This section described the participants’ concern of failing to view and learn through online-recorded lectures due to procrastination, which is an intrinsic human characteristic.

## Quantitative and qualitative findings showing the contribution of live lecture in comparison to recorded lectures and student reaction to live lectures

### Quantitative findings-4

**Figure 5.**
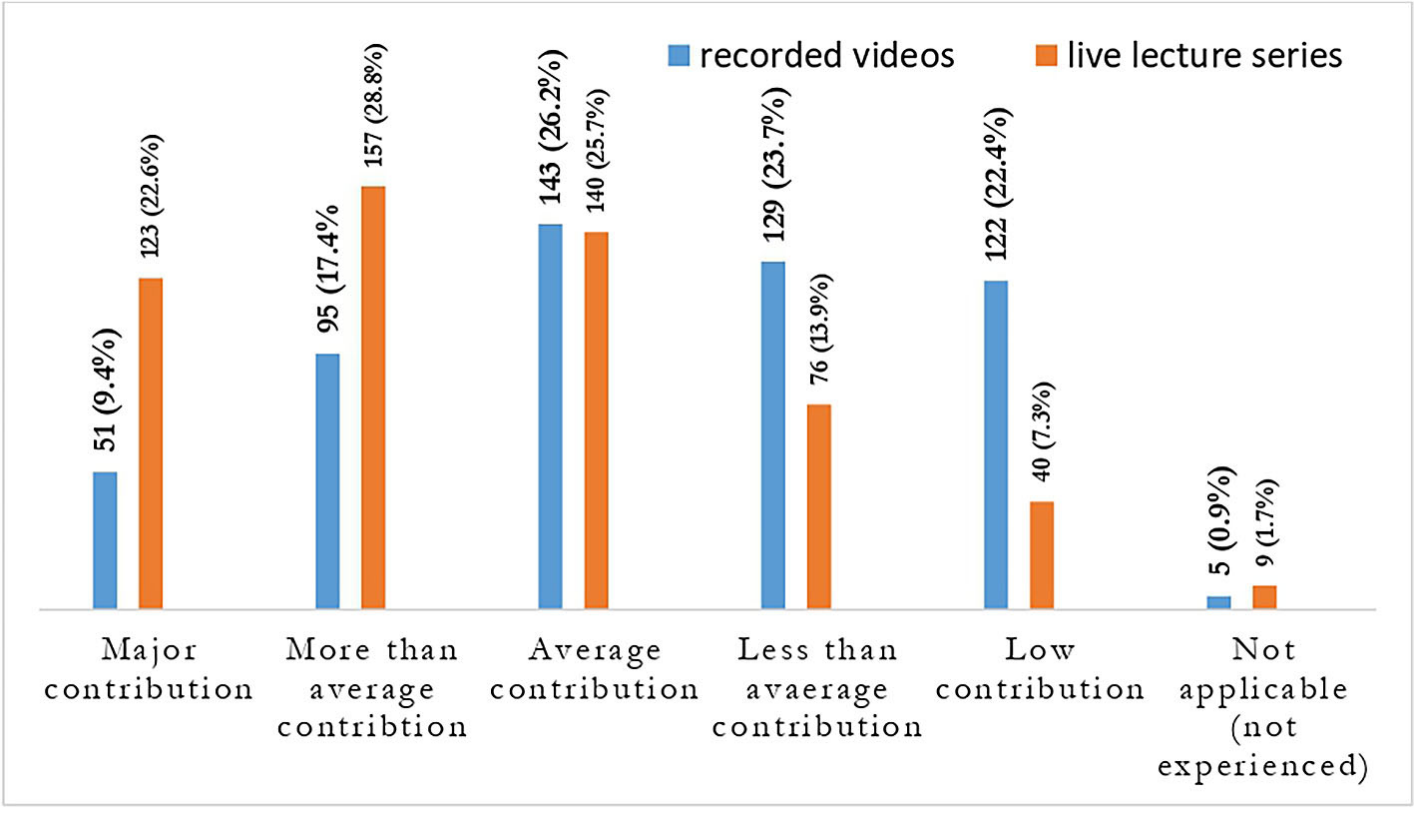
Students response showing the contribution of live lectures in comparison to recorded lectures.

**Figure 6.**
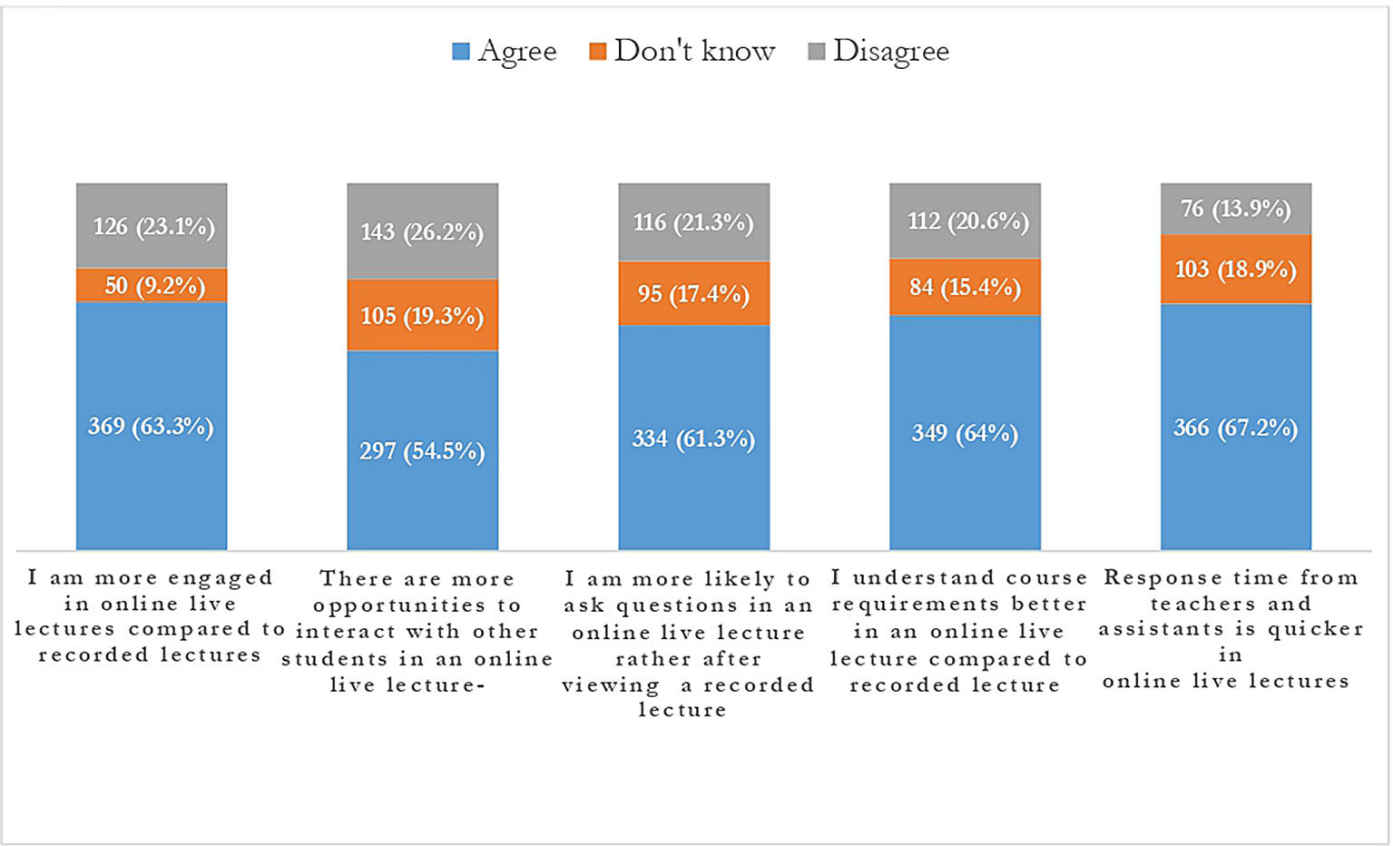
Students’ reaction to live lectures.

The quantitative data clearly shows that the live lecture series’ have a relatively higher percentage of contribution 123 (22.6%) towards learning in comparison with instructor recorded video lectures 51 (9.4%).

Also, most of the students 334 (61.3%) agreed that they were more likely to ask questions in online live lectures and response time from teachers was quicker in online live lectures 366 (67.2%). The opportunity to interact with other students is another feature liked and agreed by students 297 (54.5%).

### Qualitative findings-4: Live lectures are superior due to better clarified concepts

Live online lectures enabled the participants to clarify their doubts. Learners had the opportunity to clarify their questions as the online platform permitted students to unmute themselves and directly communicate with the instructor or post questions in the chat boxes and interact. A student from FGD 3 says, “ *We can ask questions personally to the Professor, that’s the most valid point I see for a live lecture*”; *“Live online lectures are better in my opinion, given that I have the opportunity to interact with the professor as and when needed”* (Open-ended responses). The online platform also enabled them to learn from peers as this was a shared online platform. “ *We can quickly move on like in live lectures per se and clarify the trivial doubts”; “… if there is a doubt that let’s say two or three other students might have had it, it gets cleared like on a shared platform”* (FGD 1).

### Mixed-methods inferences 4: Confirmation

Quantitative findings revealed that the online live lectures helped them to understand the course material better and there was a possibility of interaction with peers and professors. Qualitative findings confirmed the opportunities for interacting with peers. Also, the teachers enabled better clarification of concepts during a live lecture. Thus, the quantitative and qualitative findings on students’ reactions towards live lectures complemented each other.

## Quantitative and qualitative findings on students’ perception towards live lectures

### Quantitative Findings-5

**Table 2.** Quantitative findings on students’ perception towards live lectures.

Statements	Agree	Don’t know	Disagree
I can manage my time as per the online live lectures as compared to recorded lectures.	281 (51.6)	106 (19.4)	158 (29.0)
I am a multi-tasker and I do multiple things while watching an online live lecture.	239 (43.9)	114 (20.9)	192 (35.2)

Quantitative data set showed better time management with live lectures, indicating the possibility of greater learning.

### Qualitative findings-5: Better time management but strict schedules in live lectures leads to lack of focus

Students experienced exhaustion at the end of the day by attending a rigid and lengthy schedule of online live classes. They also lack concentration by attending long live online lectures. Difficulty in concentration was experienced in the online live lectures with distractions created in between the classes. A student from FGD 2 says, *“…I feel it brings down a lot of my concentration, so I tend to drift off very fast and very quickly and the smallest of things can distract me when I’m listening to an online lecture."* A similar experience was shared in FGD 3 and 4 *“We tend to be distracted a lot and when we have live lectures, we might have some other duties” (*FGD 3); “ *However, six hours of continuous classes is tiring and monotonous”; “Limited lectures should be held to avoid saturation*” (Open-ended responses).

### Mixed-methods Inferences 5: Discordance

Quantitative and qualitative findings on the perception of students towards live lectures were discordant, as quantitative findings show better time management with live lectures, indicating the possibility of better learning. However, qualitative findings showed probabilities of distraction and the likelihood of inadequate learning. Though students perceive that the online live lectures create a schedule for better time management, a rigid and long lecture schedule in a day leads to a lack of focus.

## Quantitative and qualitative findings on students’ preference towards a variety of learning resources

### Quantitative findings-6

**Figure 7.**
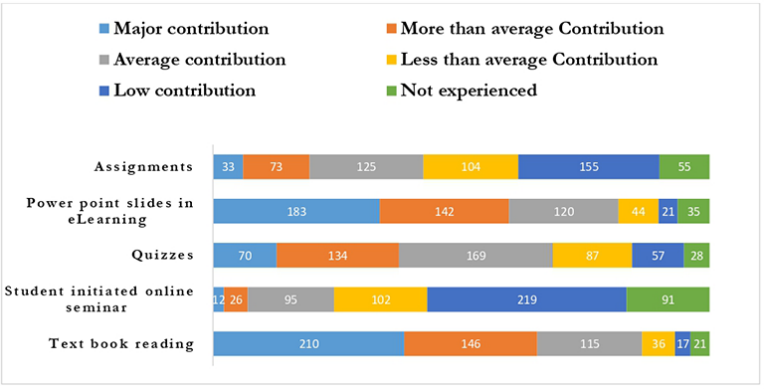
Preference of students towards variety of learning resources.

Out of the variety of learning tasks offered, students perceived that the textbooks, quizzes, and PowerPoint slides’ contribution were average and above i.e.,471 (86.4%), 373 (68.4%), and 445 (81.7%) respectively in comparison with student-initiated seminars 133 (24.4%) and assignments 231 (42.4%).

### Qualitative findings-6: Quizzes and PowerPoints supplement learning

Students perceive that for their learning, the quizzes and PowerPoint slides contribute largely when compared to the student-initiated seminars and assignments. A student from FGD 1 said, *“Yes, definitely, like small quizzes, multiple choice questions at the end of the physiology lectures. They do help us”* (FGD 1). There was a request from students to have more quizzes for better clarification. *“…if you're explaining some concept and in between, you give MCQ case scenario then that'll encourage us to think about the concepts and understand it better*” (FGD 4). Students also demanded to share the PowerPoint slides for better learning. *“… if the lecture is supplemented with handouts or PPT is given by the teachers that would help us learn better”* (FGD 4). Conversely, inattentiveness towards student-initiated seminars and assignments was expressed. *“Assignments, students just copy and paste”.* *“… most of them do not listen to the student seminar. Most of the students do not listen to other students’ presentation”* (FGD 4).

### Mixed-methods inferences 6: Confirmation

Quantitative and qualitative findings complemented each other, with the preference towards quizzes and PowerPoint slides than assignments and student-initiated seminars.

## Quantitative and qualitative findings on students’ reaction to online lectures after the pandemic

### Quantitative findings-7

**Figure 8.**
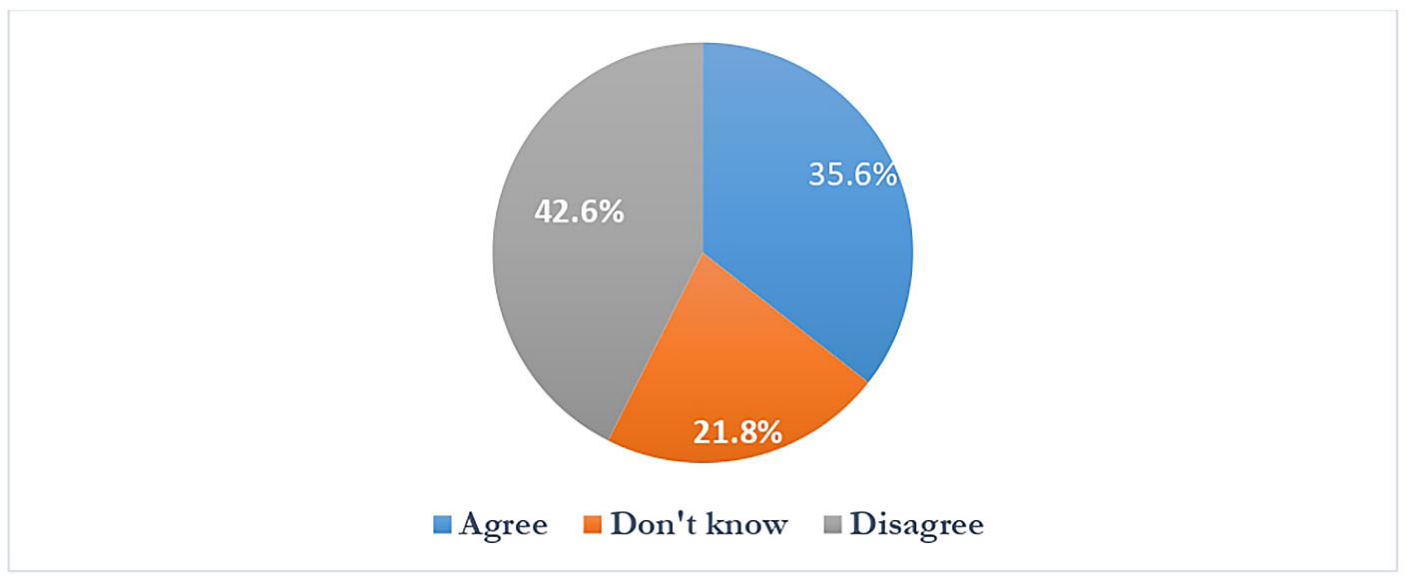
Students reaction for online lectures after the pandemic.

Students either disagreed or were inconclusive 351 (64.4%) about the online classes continuing after the pandemic.

### Qualitative findings-7: Preference for classroom (face-to-face) learning

Students perceived that the physical presence of the teacher is essential for effective learning. The teacher’s presence in traditional didactics keeps the students more attentive and motivated for enabling them to note the extra lecture points during the in-person classes. Face-to-face classes in traditional classrooms also facilitate clarification of difficult concepts, peer interaction and intensify the learning. The supporting verbatim from the students are, *“…when we were there in University campus, for the face-to-face lectures, we were more attentive rather than here at, you know, hometown”*(FGD 1); *“…I would personally prefer class teaching because I find a lot of distractions during the video lectures”, “I also think that having class room lectures is better because I am forced to sit down and have to concentrate on what’s happening”; “After we come back there, if the option is given, I think traditional teaching would be enough”; “… teaching you in person, getting it practiced in front of them, then correcting your mistakes all of that stuff is like very important when you’re learning clinical skills”* (FGD 2); *“It’s like now that we are at home, so we don't tend to study. Uh, we are all like relaxed”; “Face-to-face lectures by teachers themselves would be really helpful to understand the topics better”* (Open-ended responses).

### Mixed-methods inferences 7: Expansion

Students prefer traditional classroom teaching in comparison to online live lectures. Quantitative findings showed students’ desire for traditional classroom classes after lifting of COVID-19 lockdown. Qualitative findings illustrated the reasons such as the importance of teacher’s physical presence for effective learning for better attention, focus, motivation, and clarifying the concepts for desiring the traditional classes after lifting of COVID-19 lockdown.

## Discussion

This mixed-methods research study integrated qualitative and quantitative factors to examine undergraduate medical students’ experiences and perceptions regarding online lectures conducted during the period of COVID-19 restrictions.

### Education continuity and COVID-19

From our study, 75.2% of students agreed that online teaching with recorded and live lectures created a definite impact on their educational experiences and conferred a sense of continuity in learning. The students were grateful for the availability of virtual classes so that they could complete their course amidst the pandemic (Qualitative findings 1). Our findings support a recent study that reported that distance e-learning delivered either as synchronous live stream, recorded lectures, or both, represented an optimal solution to maintain continuity of medical education ( Al-Balas *et al.*, 2020).

### Challenges and benefits of recorded lectures

Recorded lectures were deployed as a method for asynchronous online learning. One of the positive features of the asynchronous availability of learning resources is that they can be reviewed by learners at their convenience. The students can go through the content and complete it based on their understanding. Learners can also adjust the viewing and pacing of recorded lectures based on their learning speeds. In our study, students who were fast learners streamed the videos at higher speeds, while slower learners paused and repeated lectures to suit their ability to absorb information, facilitate note-taking and refer to recommended learning resources such as textbooks (Qualitative findings 2). In an online learning environment, the learners who are not ready to organize and control their learning process have been shown to yield to academic procrastination and poor performance ( Hasan *et al.*, 2021). Although students expressed that the recorded lectures were important, the tendency to procrastinate and lack of discipline resulted in the majority of students (60.9%) watching less than 50% of recorded lectures (Quantitative findings 3). A previous study conducted in an emergency medicine course reported that 70% of students did not use asynchronous material during their clerkship ( Lew and Nordquist, 2016).

### Live lectures

The participants in our study regarded live online lectures to be superior compared to recorded lectures for a better understanding of course concepts (Qualitative findings 4). The learner feels more attentive when the teacher is directly communicating with students and 63.3% of students agreed that they were more engaged during live lectures (Quantitative findings 4). Synchronous communication with live lectures also enables students to interact, pose questions and receive a fast response from teachers. Our study participants also elaborated that during a live lecture, peers raise questions that inspire more discussions and there is a shared learning experience.

Cognitive presence as per CoI framework relates to the attainment of course objectives predominantly supported by critical thinking. Social presence refers to faculty-student and peer interactions that would establish a community of learners for peer learning. The instructor presence supports and interlinks cognitive and social presence ( Stodel *et al.*, 2006). Through the lens of this theoretical framework, the enhanced ability to understand and reflect on course requirements with online live lectures and not after recorded lectures signifies that learners perceive higher cognitive presence with live lectures ( Kang *et al.*, 2007). The in-depth discussions facilitated by the instructor during online live lectures promote cognitive presence. Also, teachers’ responsiveness to students’ needs and timely feedback improves the social presence in online learning. Learning is most effective in an online learning environment when peer collaboration occurs. The teacher facilitates collaborative learning and the shared learning experience, as voiced by one of the participants, fosters the social presence construct.

Online live lectures are not without their challenges and drawbacks. Our study participants had mixed responses regarding the online live lecture structure and schedule. While students opined that the live lectures provided them with a schedule for better time management, they expressed difficulty in concentrating for continuous online classes throughout the day and that fatigue due to online classes compromised their reserve for self-study (Qualitative findings 5). Stodel E J *et al.* reported that the inclusion of synchronous components in online learning takes away the very features that attracted many learners to this mode in the first place: the convenience, and flexibility afforded by not having to meet at a specific time or for a certain duration ( Stodel *et al.*, 2006). If students have to meet at a given time, they may not find this flexible enough. Despite being well-received by students, a recent qualitative study published on the sudden transition to the synchronized format during the COVID-19 pandemic reported that medical students disliked attending a lot of lectures in one day and perceived adjustment issues related to the duration and arrangement of online sessions ( Khalil *et al.*, 2020). External barriers such as technical issues (internet speed and connectivity) can pose a challenge to synchronized student learning ( Adedoyin and Soykan, 2020). Having limited capacity with only one speaker able to speak at a time can mean that discussions take longer, and students have to wait for their turn, especially in large virtual classrooms ( Redmond, Parkinson, Mullally, 2007). In the context of an online learning environment, students have to take initiative. When there is an absence of discipline enforced in face-to-face classes, individual motivation becomes key to determining success ( Daspit *et al.*, 2015).

### Student learning resource preferences

Students require a variety of support from instructors for effective learning. In addition to providing curated digital resources, periodic online quizzes created by faculty for formative assessment facilitates practice and self-review for learners ( Cohen and Sasson, 2016). Supplementary material provided in the form of PowerPoint slides and detailed notes has been shown to enhance understanding of core concepts. A survey conducted by Abdulla HA *et al.* revealed positive student perception with supplementary aids mainly because it served as a revision tool and it reduced the time required to search relevant information ( Abdulla and O’Sullivan, 2019). In our study, students expressed a preference for online quizzes and mentioned PowerPoint slides and handouts as useful resources for learning. Also, there was low contribution in assignments aimed towards learning. The tendency to delay the task leads to poor quality and accuracy due to pressure associated with completing the assignment against a deadline ( Santelli *et al.*, 2020).

### Preference for face-to-face classes in a traditional classroom setting

Although medical educators contemplate online learning to continue in some shape and form in the future, participants from our study disagreed or were inconclusive about the preference for online lectures continuing after the COVID-19 pandemic. The psychological impact of classrooms during in-campus teaching has an enormous influence on learning according to the students in our study (Qualitative findings 7). As per the opinion of students from Rehana *et al.*, college campus and clinics are most suitable for learning because it provides them with special learning environment ( Khalil *et al.*, 2020). Similar to the findings from our study, a recent survey conducted in Jordan on medical students reported a low level of satisfaction with distance e-learning, but they expect distance learning to replace traditional class learning for theoretical knowledge ( Al-Balas *et al.*, 2020). Medical education is unique in that it requires training in clinical skills using skills lab and hospital settings during clerkships and this component of training got significantly compromised during COVID-19. The students expressed interest in returning to campus to resume hands-on training and practice under the supervision of faculty (Qualitative findings 7).

This study is characterized by few limitations that are worth considering for future research. Although our study design had a quantitative segment supported with a validated questionnaire, caution needs to be ascertained while generalizing the results as this study was carried out at a single teaching institute. It would be worthwhile to collect data from several centres and do a comparative study. Though the study involved a significantly large sample size of students, the low response rate for the survey is a limitation. This study only included student reactions to online learning, leaving out perceptions of faculty and administrators. Due to time constraints, not all stakeholders could be included in this mixed-methods study. Future research designs can incorporate dimensions from faculty with views and issues related to the design and delivery of online teaching. Extending the CoI framework, researchers are encouraged to explore how presences other than the cognitive, instructor and social presences would relate with an online educational context. With online teaching, and learning with digital resources transforming the landscape of medical education, future research can aim to explore the impact of blended learning designs.

## Conclusions

With the onset of the COVID-19 pandemic, in-person classes were cancelled and the medical schools had to adopt newer modalities of teaching and learning such as distance learning and remote asynchronous learning. In our study, pre-recorded lectures were made available to students to learn asynchronously and synchronous online live lectures were conducted during COVID-19 restrictions. In response, the medical students reported that online learning supported continuity in medical education during the COVID-19 pandemic. Online live lectures compared to recorded video lectures could foster greater cognitive, social, and instructor presences and hence live lecture series were perceived by students as superior with a major contribution towards learning. The instructor presence is crucial in an online learning environment and the students valued instructor interaction in online live lectures. Faculty integrate social presence in online live lectures by facilitating peer learning and creating a shared learning experience. Students appreciated the recorded lectures for their convenience and flexibility for asynchronous learning. But regarding preferences for their future, students strongly proposed face-to-face teaching and regarded the learning environment on the campus an effective educational experience.

### Recommendations

Successful online learning requires student-centric design allowing learner flexibility and promoting peer collaboration. The pedagogy for online learning has to be redesigned embedding principles of instructional design and online technologies. Digital resources developed can include bite-sized e- learning resources and micro-learning nuggets for personalized learning and boosting student engagement. Modules for self-learning can be considered with questions for formative assessment and inbuilt inline remediation. The synchronized real-time lectures must be outlined with concise, clear objectives and should stimulate higher-order learning. The asynchronous component should be supported by the robust learning management system to facilitate storage of course materials and sharing, promote the development of a community of learners through discussion forums and conduct periodic assessments and assignments. Efforts must be made by institutes to educate students regarding plagiarism in assignments and inculcate a sense of responsibility among learners.

Since clinical training is essential in medical education, measures have to be taken to address certain specific components of clerkship online such as teaching communication and clinical reasoning, exposing students to virtual ward rounds. The faculty training for creating and using digital resources have to be undertaken to prepare teachers for remote and blended learning that can be envisioned as the future of medical education.

## Data availability

### Underlying data

Figshare: Data on perceptions of students towards online learning in medical education. https://doi.org/10.6084/m9.figshare.20171264.v2 ( Kirtana *et al.*, 2022).

This project contains the following underlying data:

- Responses of medical students to survey on online learning.csv
- FGD Transcript student perceptions on online learning.pdf

### Extended data

Figshare: Data on perceptions of students towards online learning in medical education https://doi.org/10.6084/m9.figshare.20171264.v2 ( Kirtana *et al.*, 2022).

This project contains the following extended data:

- Questionnaire & FGD guide.pdf

are available under the terms of the Creative Commons Attribution 4.0 International license (CC-BY 4.0).
