# Supplementary material for: Student perceptions towards online learning in medical education during the COVID-19 pandemic: a mixed-methods study
Source: F1000Res. 2022 Aug 25;11:979. [Version 1] doi: 10.12688/f1000research.123582.1 (PMC9880340; doi:10.12688/f1000research.123582.1)
Supplement: Peer review history

# Peer review: Reviewer response for version 1

**DOI:** 10.5256/f1000research.135702.r159924

**Published:** 2023-01-26

**Authors:** Forman Erwin Siagian

**Affiliations:** 1 Department of Parasitology, Faculty of Medicine, Universitas Kristen Indonesia, Jakarta, Indonesia

To my opinion, this study revealed the most recent situation that most higher education institution faced, especially faculty of medicine with its own uniqueness. These researchers use undergraduate medical students as subjects who are directly affected by the changes caused by Covid and how they modify their way of learning and shifted it from class based to online.

 This article is neatly written that analyzing the implementation of online learning/teaching made available by the institution for their students.

 in my observation, this article shows positive values ​​that is in context of:

1. The 1197 words that make their introduction described extensive background of their situation and how they modify their methods; and this become the gap that the researchers would like to explore, 
2. A simple but yet comprehensive study design has been used, combining both quantitative and qualitative approach with sufficient number of respondents.
3. Methodologically correct and straight to the point analysis make the result easy to understand
4. The Discussion section contain not only the supporting data from similar previous studies that were conducted elsewhere, but also limitations of the study and future prospect of continuing this study more deeply.

it would be more balanced if I also conveyed some of the limitations (in my personal perspective):

1. There is no information on student positions, whether far or close to campus. Because in my opinion this is very likely to influence their perceptions of online learning activities
2. The number of FGD participants was very small (compared to the number of survey respondents) so there could be a possibility of bias (for example the FGD participants were 'carried away' by the FGD leader)

But overall this article is good and relevant to current conditions and can be part of learning for other medical education institution

Is the work clearly and accurately presented and does it cite the current literature?

Partly

If applicable, is the statistical analysis and its interpretation appropriate?

Yes

Are all the source data underlying the results available to ensure full reproducibility?

Yes

Is the study design appropriate and is the work technically sound?

Yes

Are the conclusions drawn adequately supported by the results?

Yes

Are sufficient details of methods and analysis provided to allow replication by others?

Yes

Reviewer Expertise:

Parasitology, Cross sectional study, Medical education

I confirm that I have read this submission and believe that I have an appropriate level of expertise to confirm that it is of an acceptable scientific standard.

# Peer review: Reviewer response for version 1

**DOI:** 10.5256/f1000research.135702.r148549

**Published:** 2022-09-07

**Authors:** Nachiket Shankar

**Affiliations:** 1 Department of Anatomy, St. John's Medical College, Bengaluru, Karnataka, India

This is a well-written article exploring the perceptions of students about the online teaching and learning that took place during the lockdown imposed during the COVID pandemic at a medical college in India. The article has many strengths which are detailed below. 

1. The introduction is comprehensive and provides appropriate background information. 
2. A suitable theoretical framework has been used to guide the study.
3. The large sample size and apt study design contribute to the methodological rigor of the study. 
4. Both the quantitative and qualitative data have been appropriately analyzed in the results section. 
5. The discussion section adequately explains the results of the study in light of the existing literature. 

A few suggestions are as follows:

1. The research question and research objectives could be explicitly stated in the introduction section. 
2. The process used to select students to participate in the focus group discussions could be described in more detail in the methods section. 
3. The description below the 2nd bar from the left in Figure 4 of the results section has a typographical error that needs to be corrected. 
4. One limitation that has been mentioned is the low response rate for the survey. The possible implications of this on the results of the study could be explained. 
5. The sentence, *"Since clinical training is essential in medical education, measures have to be taken to address certain specific components of clerkship online such as teaching communication and clinical reasoning, exposing students to virtual ward rounds." *in the recommendations sub-section could be omitted as it contains information that is not related to the current study.

Is the work clearly and accurately presented and does it cite the current literature?

Yes

If applicable, is the statistical analysis and its interpretation appropriate?

Yes

Are all the source data underlying the results available to ensure full reproducibility?

Yes

Is the study design appropriate and is the work technically sound?

Yes

Are the conclusions drawn adequately supported by the results?

Yes

Are sufficient details of methods and analysis provided to allow replication by others?

Yes

Reviewer Expertise:

Medical education and anatomy

I confirm that I have read this submission and believe that I have an appropriate level of expertise to confirm that it is of an acceptable scientific standard.
